# Supplementary material for: Immunological Responses to Seoul Orthohantavirus in Experimentally and Naturally Infected Brown Rats (Rattus norvegicus)
Source: Viruses. 2021 Apr 12;13(4):665. doi: 10.3390/v13040665 (PMC8070117; doi:10.3390/v13040665)
Supplement: Supplementary file 1 [file viruses-13-00665-s001.pdf]

Supplementary materials

Table S1. Sex difference in antibody production against SEOV in wild rats.

	**IgM**	**IgG+**	**IgG-**	**Total**
Male	+	13	0	13
	-	7	79	86
	total	20	79	99
Female	+	15	1	16
	-	8	76	84
	total	23	77	100

Table S2. SEOV genome detection from sera of IgG-positive rats

**Viral Genome in Serum**	**IgM Antibody in Sera**		
	+	-	
+	8	2	$p = 0.39$
-	17	9	

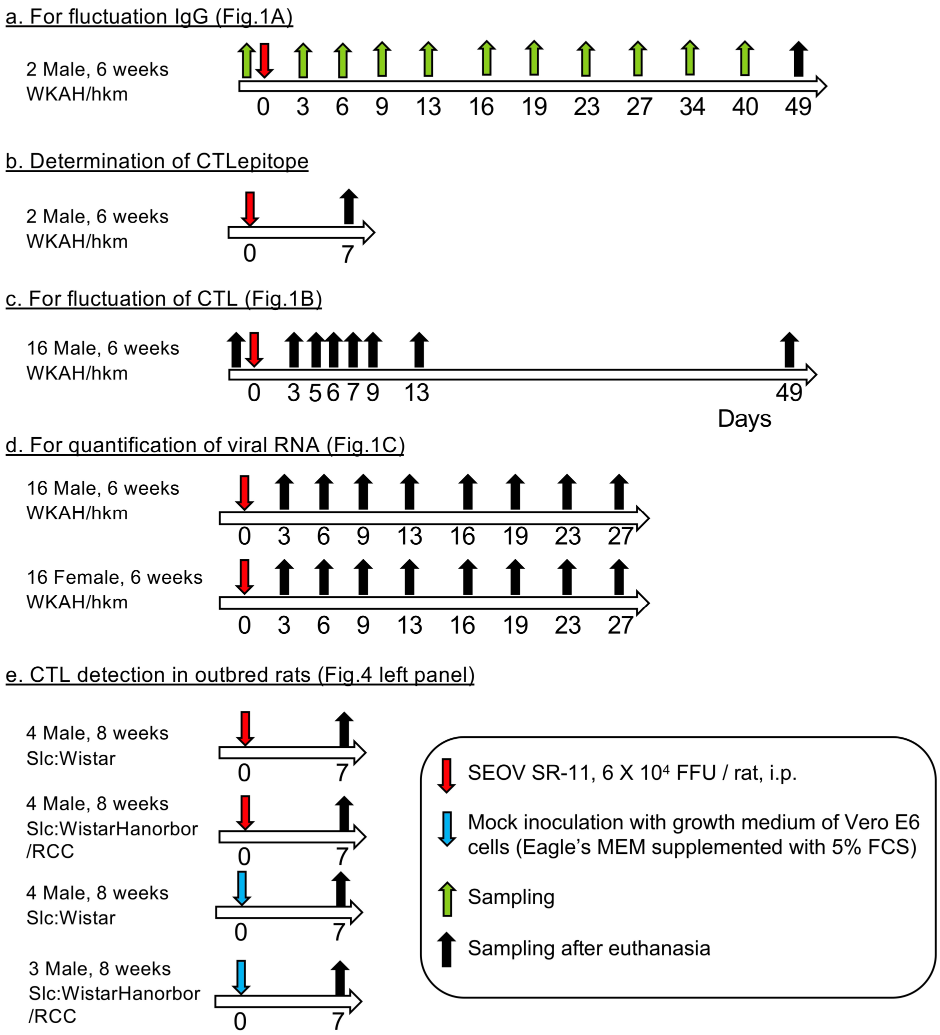

Supplementary figure 1:Sample collection from laboratory rats experimentally inoculated with SEOV SR-11

Figure S1.: Sample collection from rats inoculated with SEOV SR-11.

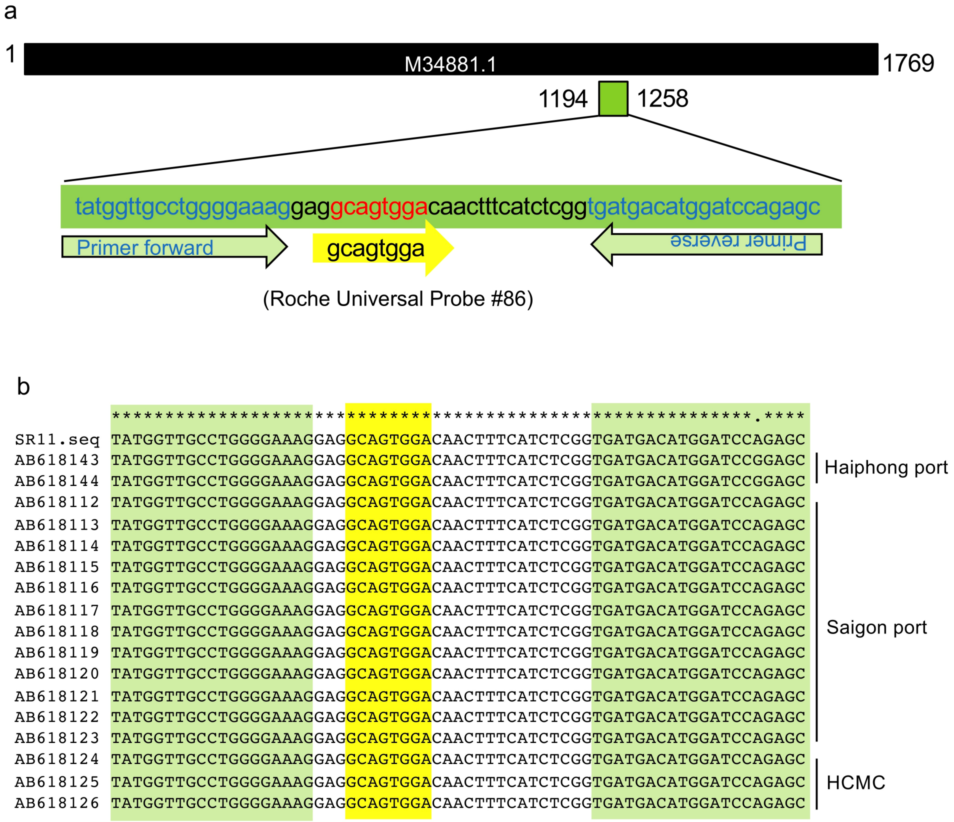

Supplementary figure 2 : Primers and a probe for quantification of viral RNA used in this study

a. Based on sequence of S genome segment of SEOV strain SR-11 (Accession no. M34881.1), ProbeFinder software (provided by Roche) recommended several sets of primers and probes. This primer set was selected because sequence of this amplicon was highly conserved among SEOV strains.
b. Nucleotide sequence similarities of Vietnamese SEOV to SR-11 were 98.5%-100%. Regions of primers and probe were shown in green and yellow, respectively.

Figure S2: Primers and a probe for quantification of viral RNA used in this study.

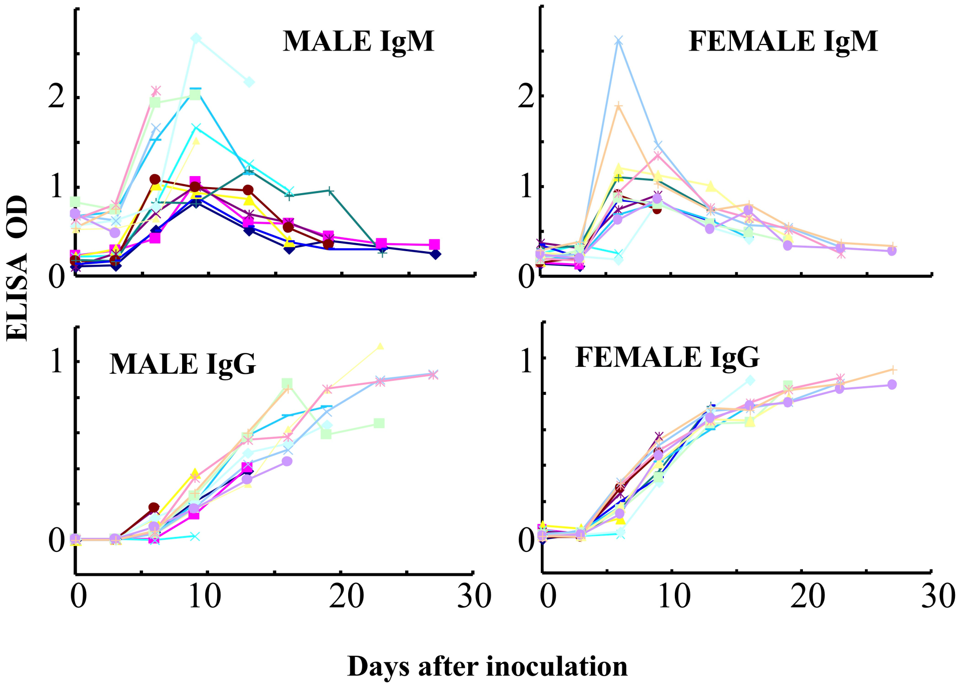

Supplementary figure 3. ELISA ODs of IgM and IgG antibodies in all rats inoculated with SEOV.

These rats were used to examine fluctuation of CTL activities and viral load in lungs, serum, and feces.

Figure S3: ELISA ODs of IgM and IgG antibodies in all rats inoculated with SEOV.
